# Transcription Factors Interact with ABA through Gene Expression and Signaling Pathways to Mitigate Drought and Salinity Stress

**DOI:** 10.3390/biom11081159

**Published:** 2021-08-05

**Authors:** Quaid Hussain, Muhammad Asim, Rui Zhang, Rayyan Khan, Saqib Farooq, Jiasheng Wu

**Affiliations:** 1 State Key Laboratory of Subtropical Silviculture, Zhejiang A&F University, 666 Wusu Street, Hangzhou 311300, China; quaid_hussain@yahoo.com (Q.H.); rui.zhang@zafu.edu.cn (R.Z.); 2 Tobacco Research Institute, Chinese Academy of Agricultural Sciences, Key Laboratory of Tobacco Biology and Processing, Ministry of Agriculture and Rural Affairs, Qingdao 266101, China; asim.ktk91@aup.edu.pk (M.A.); rayyanswb@gmail.com (R.K.); 3 Guangxi Key Laboratory of Agric-Environment and Agric-Products Safety, Agricultural College of Guangxi University, Nanning 530004, China; saqibhort@gmail.com

**Keywords:** ABA, drought, genetic engineering, pathways, salinity, transcription factors

## Abstract

Among abiotic stressors, drought and salinity seriously affect crop growth worldwide. In plants, research has aimed to increase stress-responsive protein synthesis upstream or downstream of the various transcription factors (TFs) that alleviate drought and salinity stress. TFs play diverse roles in controlling gene expression in plants, which is necessary to regulate biological processes, such as development and environmental stress responses. In general, plant responses to different stress conditions may be either abscisic acid (ABA)-dependent or ABA-independent. A detailed understanding of how TF pathways and ABA interact to cause stress responses is essential to improve tolerance to drought and salinity stress. Despite previous progress, more active approaches based on TFs are the current focus. Therefore, the present review emphasizes the recent advancements in complex cascades of gene expression during drought and salinity responses, especially identifying the specificity and crosstalk in ABA-dependent and -independent signaling pathways. This review also highlights the transcriptional regulation of gene expression governed by various key TF pathways, including AP2/ERF, bHLH, bZIP, DREB, GATA, HD-Zip, Homeo-box, MADS-box, MYB, NAC, Tri-helix, WHIRLY, WOX, WRKY, YABBY, and zinc finger, operating in ABA-dependent and -independent signaling pathways.

## 1. Introduction

Being sessile, plants are susceptible to various adverse environmental conditions. Plants inherently live in harsh conditions [1], and the natural environment of plants comprises abiotic and biotic stressors [2]. Abiotic stressors are the foremost limiting factors, e.g., drought, high salinity, low temperature, high temperature, nutrient stress, and heavy metals, and are hostile to plant growth and development, ultimately affecting crop productivity and sustainability [3,4,5].

Drought and salinity periods interrupt the ionic and osmotic strength, encourage the redox balance and cellular energy, and cause the loss of photosynthesis [6]. Drought stress is one of the leading aspects of regulating crop production, provoking many physiological, molecular, biochemical, and anatomical changes [7]. Salinity is a significant factor that decreases crop production by deteriorating plant health [8]. There are different transcription factors (TFs) involved in drought and salinity stress responses; for example, *MtHB2* in *Medicago truncatula* [9], *Zmhdz10* in maize [10], *OsGATA23a* in rice [11], and *ATHB17* in Arabidopsis [12] play an essential role in response to drought and salinity stress. Phytohormones are crucial integrators for the association and growth of adaptive mechanisms in response to stress. Abscisic acid (ABA) is a significant regulator of numerous flexible traits of plant developmental improvements, including embryo maturation, germination, seed dormancy, floral initiation, and root growth. ABA also decreases the detrimental effects of stress, such as those caused by drought, in plants [13].

ABA is a plant hormone that helps plants respond to drought. Drought-responsive genes may be divided into two categories based on their ABA response: ABA-dependent and ABA-independent genes [13]. Even though numerous drought-responsive genes are engaged in the ABA signaling system, most drought-induced genes do not react to ABA treatment, indicating the presence of ABA-independent drought-response pathways [14]. Numerous genes are involved in response to drought and salinity stress; under such conditions, tolerance is triggered by osmotic stress, which liberates ABA [15]. ABA-dependent and -independent mechanisms control osmotic stress-responsive gene expression [16]. Plants’ stress response systems, for example, comprise both ABA-dependent and ABA-independent activities. *DREB2A/2B*, *AREB1*, *RD22BP1*, and *MYC/MYB* are the TFs that interact with their corresponding cis-acting elements, DRE/CRT, ABRE, and MYCRS/MYBRS, respectively, to regulate the ABA-responsive gene expression [17].

DFREB1/CBF-type TFs are critical in water and salt stress tolerance in higher plants. These TFs regulate the expression of target genes by binding to *CRT/DRE* sites in their promoters. Drought and salt stress, as well as exogenous ABA, stimulated *MbDREB1* expression [18]. *MbDREB1* promoter analysis identified an ABA-responsive element (ABRE) that induced an ICE1-like binding site, two MYB recognition sites, and three stress-inducible GT-1 boxes. ABA, drought, and salt treatments activated GUS activity in transgenic Arabidopsis [18].

Conversely, both ABA-independent and ABA-dependent stress-induced genes (*COR15a* and *rd29B*, respectively) were upregulated in Arabidopsis overexpressing *MbDREB1*. Both ABA-dependent and ABA-independent pathways used *MbDREB1* to activate plant tolerance to low temperature, drought, and salt stress [18]. Salt and drought stress induced *PR-1*, *PR-5*, *RAB-18*, and *RD-29A* genes in plants pretreated with ABA [19]. Both ABA-dependent and ABA-independent osmotic stress signaling first adjust constitutively expressed TFs, leading to the expression of early response transcriptional activators, activating downstream stress tolerance effector genes [20]. TFs play diverse roles in controlling gene expression [21] in plants, necessary for regulating biological processes, such as development and environmental stress responses [22]. TFs are the key molecular switches that enable plants to withstand harsh conditions and direct the plant’s developmental process in response to abiotic stress [23]. TFs play an essential role in crop improvements and are considered good candidates for improving tolerance to various abiotic stressors [24]. TFs are considered the best genetic materials to breed and develop stress-tolerant crop varieties because of their role as master regulators of many stress response-related genes compared to manipulation in a single functional gene [25]. For improving abiotic stress resilience in plants, Tripathi et al. [26] discussed the contributions of new technologies such as DAP-seq, bulk, or single-cell ChIP-seq RNA-seq yeast 1-hybrid and CRISPR/Cas9. ChIP-seq is a method used to analyze protein interactions with DNA. ChIP-seq combines ChIP with massively parallel DNA sequencing to identify the binding sites of DNA-associated proteins [26]. The chIP or yeast one-hybrid method has a role in identifying co-regulated genes that are strongly differentially expressed in response to the stress treatment and characterization of TFs that regulate many target genes [26]. A high-throughput TF binding site discovery method using genomic DNA in vitro can quickly identify target genes that directly bind downstream transcription factors. The DAP-seq method is fast, inexpensive, and more easily scaled than the ChIP-seq method [27]. For example, comparative transcriptomics informed by phylogenetic relationships uncovered lineage and extremophile-specific differences in ABA response. DAP-Seq was utilized to establish GRNs in each species for the entire ABA-AREB/ABF clade [28]. The stress-inducible CRISPR/Cas9 is a robust, practical, and helpful approach for developing crop varieties resistant to climate change. It will be a helpful tool for capable and particular genome editing in different plants for several traits, including abiotic stresses [29]. Therefore, some genes are targeted through genome editing based on CRISPR/Cas9 technology in different crops. Another yeast two-hybrid method is a well-established genetics-based system that uses yeast to display binary protein-protein interactions [30]. Using such techniques, the PYL6 and MYC2 interact, and their interaction is enhanced in the presence of ABA [31].

Both drought and salinity are among the most severe abiotic factors restricting plant growth and yield. ABA-dependent (drought-inducible genes were clarified upon their induction by exogenous ABA, which means two different systems in stress-inducible gene expression; later, ABA-dependent genes are regulated by endogenous ABA based on mutant analyses) ABA-independent pathways regulate numerous genes that function in drought response. Many signaling molecules, such as ABA, ROS, H_2_O_2_, NO, Ca^2+^, PAs, and others, have been well known and revealed in plant signaling perception and transduction pathways. Many drought- and salinity-responsive genes are involved in ABA-signaling pathways, such as ABA-inducible (ABA-inducible genes were genes induced by exogenous ABA treatment in the early phase of research; now, ABA-inducible genes are regulated by endogenous ABA, as well), ABA-sensitivity (ABA-sensitivity is phenotypes related to ABA sensitive responses during germination, stress response, and so on; various mutants and ecotypes have been reported to be ABA-sensitive ones based on their responses to ABA), and ABA-mediated (ABA-mediated genes are genes regulated by ABA signaling pathways in stress responses and plant growth). However, several drought- and salinity-induced genes do not respond to ABA signaling, showing that ABA-independent signaling pathways also regulate the response to drought and salinity stress. This review mainly focuses on the recent progress and development of TFs and their upstream and downstream ABA-related genes to emphasize the role of ABA genetic engineering in drought and salinity tolerance in various crops and sheds light on various TF families’ functions to orchestrate the tolerance response in crop species.

### TFs Regulatory Network in Response to Drought and Salinity Stress

**Figure 1:**
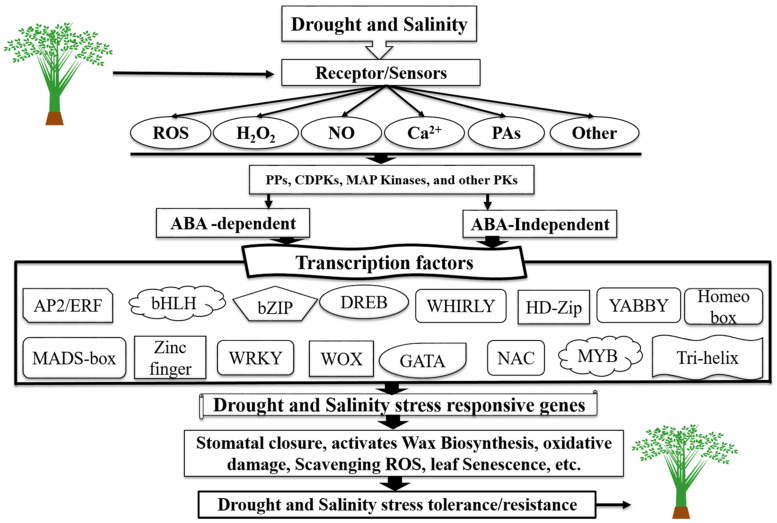
Schematic diagram of TFs as key components in transcriptional regulatory networks during drought and salinity stress-signaling pathways in different crops/plants. A diagrammatic representation of gene expression and drought and salinity stress signal perception in plants via ABA-independent and ABA-dependent pathways (modified from Khan et al. [34]).

A transcription factor is a protein that binds to DNA and regulates gene expression by promoting or suppressing transcription. The function of TFs is to regulate turn on and off genes and ensure their expression in the suitable cell at the right time and the right amount throughout the life of the cell and the organism [32]. Transcription factors are modular in structure and contain two domains. The first is the DNA-binding domain, which attaches to specific DNA sequences (enhancer or promoter) adjacent to regulated genes. DNA sequences that bind transcription factors are often referred to as response elements [33]. The second is the activation domain, which contains binding sites for other proteins such as transcription coregulatory. These binding sites are frequently referred to as activation functions, transactivation domains, or trans-activating domains but do not mix with the topologically associating domain [32,33]. Generally, a stress signal transduction pathway comprises the following key steps: (1) signal perception, (2) signal transduction, and (3) stress response. The first step in activating a signaling cascade for drought and salinity stress recognizes stress signal via receptors located on the membrane of the plant cell [34]. After recognition, these sensors transmit the signal downstream through phytohormones and second messengers such as Ca^2+^ and ROS [35]. The second messengers, such as ROS, trigger another set of ROS-modulated PKs and PPs, including MAPK cascades, CDPKs, CBLs, CIPK, and many other PKs, as well PPs such as some PP2Cs (Figure 1) [34]. ABA is the principal hormone involved in the coordination of abiotic stress in plants [36]. The different stress tolerance responsive TFs usually function independently. However, there is a possibility that some level of cross-link occurs between these TFs (Figure 1). The last step is the expression of functional genes involved in different functions such as stomatal closure, oxidative damage, leaf senescence, or indirectly regulating regulatory genes contributing to signaling cascades and transcriptional regulation of gene expression [34,37]. These abilities allow them to be excellent candidate genes for genetic manipulation of complex stress tolerance traits [38]. To date, based on genome-wide analysis, a great deal of TFs belonging to different families, such as AP2/ERF, bHLH, bZIP, DREB, GATA, HD-Zip, Homeo-box, MADS-box, MYB, NAC, Tri-helix, WHIRLY, WOX, WRKY, YABBY, and zinc finger, and so on, have been identified in different plant species [39,40].

## 2. TFs Involved in Drought and Salinity Stress Responses

### 2.1. AP2/ERF

**Figure 2:**
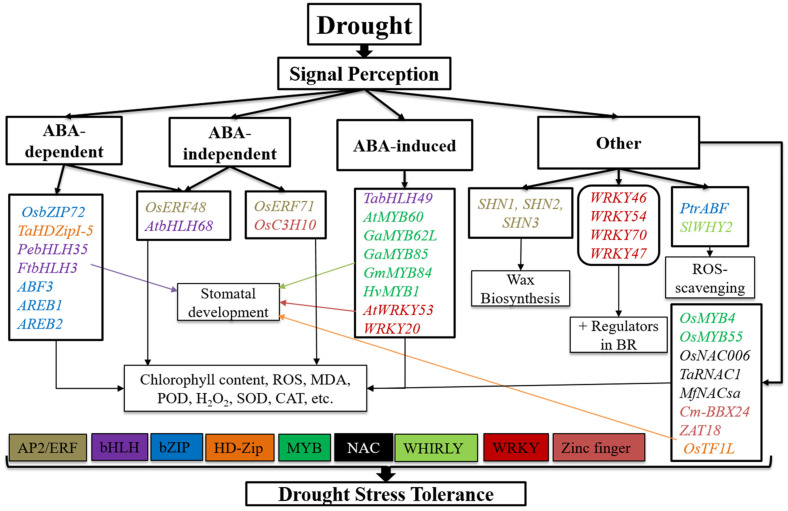
Genes and signaling pathways that regulate drought stress tolerance. These pathways include the AP2/ERF, bHLH, bZIP, HD-Zip, MADS-box, MYB, NAC, WHIRLY, WRKY, and zinc finger. These regulators control drought stress tolerance through ABA-independent and -induced pathways, which play an essential role in ROS-scavenging pathways. They are positive regulators in the BR pathway, enable wax biosynthesis and stomatal development, and alter chlorophyll, MDA, POD, SOD, and CAT content. Different text colors represent different transcription factors.

The AP2/ERF is one of the largest families of TFs, with 140–280 members in several plants [41,42], which regulate multiple responses such as stress, metabolism, and development in plants [43]. In the past, AP2/ERF genes were considered plant-specific, but recently, this domain was reported in non-plants, such as in the protists, ciliate, cyanobacterium, and phages [44,45]. The rice ABA-independent gene *OsERF48* directly binds to the promoter of *OsCML16* via AP2/ERF cis-acting regulatory elements, thereby activating its transcription. Overexpression of *OsERF48* causes regulation of *OsCML16*, a calmodulin-like protein gene that enhances root growth, drought tolerance, and grain yield and is involved in cell wall proteins, carbohydrate metabolism, and stress signaling in drought conditions in the field [46]. The rice *OsERF71* gene is an AP2/ERF TF involved in an ABA-independent pathway controlling drought resistance by regulating cell wall modifications. After *OsERF71* overexpression, roots are sufficient for drought resistance phenotypes and increase yield under drought stress [47]. The Arabidopsis *shine* (*SHN*) clade of the AP2 domain TFs activates wax biosynthesis and lipid biosynthetic pathways. Overexpression of each of the three *SHN-1*, *-2*, and *-3* genes produced a phenotype similar to that of the first *SHN* mutant. *SHN* gene overexpression changed leaf and petal epidermal cell structure, trichome number and branching, and stomatal index. The *SHN* clade plays a role in plant protective layers; for example, those shaped during abscission, dehiscence, wounding, and diverse functions are mediated by regulating lipid or cell wall components [48] (Figure 2, Table 1). The *OsERF922* gene was strongly induced in an ABA-signaling pathway after salt treatment and has been targeted successfully in rice. After overexpressing this gene, the ratio of Na^+^/K^+^ in the shoots increased, and consequently, the tolerance to salt stress decreased. The cis-regulatory sequences of the *OsERF922* gene’s GCC box (AGCCGCC) function as negative regulators of salinity tolerance by providing binding sites for particular TFs. These cis-regulatory sequences could serve as a suitable target for creating nucleotide level mutations using recent genome editing tools that improve the tolerance to salinity stress in crops [49]. AP2/ERFs stand out among the essential TFs that regulate reactions, such as metabolism, stress, and improvement in plants. *PsAP2* was isolated from a different *AP2/ERF* in *Papaver somniferum*, upregulated in response to methyl jasmonate, wounding by ethylene, and activation of ABA [43]. *PsAP2* overexpression in transgenic tobacco plants showed increased tolerance to both abiotic and biotic stress [43]. ERF TFs are involved in regulating gene expression under biotic and abiotic stress. Transcription of the *T. aestivum* ethylene-responsive factor 1 (*TaERF1*) gene was induced not only by salinity, exogenous ABA, drought and low-temperature stress, salicylic acid, and ethylene, but also by infection with *Blumeria graminis* f. sp. *tritici*. Moreover, *TaERF1* overexpression activated stress-related genes, including *PR* and COR/RD genes, under normal growth conditions and enhanced pathogen and abiotic stress resistance in transgenic plants [50] (Table 1).

### 2.2. bHLH

Basic helix-loop-helix (bHLH) TFs are involved in various developmental processes and respond to biotic and abiotic stress. Arabidopsis *AtbHLH68* encodes a bHLH through ABA-dependent or -independent pathways and is highly expressed in the lateral root, during LR elongation, and in drought stress knock-out mutants, which have development phenotypes compared to the wild type. After overexpressing *AtbHLH68*, lateral root formation was defective, and the plant had a significantly increased tolerance to drought stress, which was likely related to its enhanced sensitivity to ABA and increased ABA content. *AtbHLH68* functions to directly or indirectly regulate ABA signaling and metabolism components, likely through an ABA-dependent pathway [51]. Overexpression of the Tartary buckwheat (*Fagopyrum tataricum*) *FtbHLH3* gene in Arabidopsis increased drought tolerance, which was attributed to lower MDA, ROS leakage, higher proline content, and photosynthetic efficiency. *FtbHLH3* is an ABA-dependent pathway and is a positive regulator of drought stress tolerance in transgenic Arabidopsis [52]. The *Populus euphratica PebHLH35* gene was induced by drought and ABA treatment. *PebHLH35* is a positive regulator of drought stress responses, influencing growth, photosynthesis, stomatal aperture, and stomatal density.

**Figure 3:**
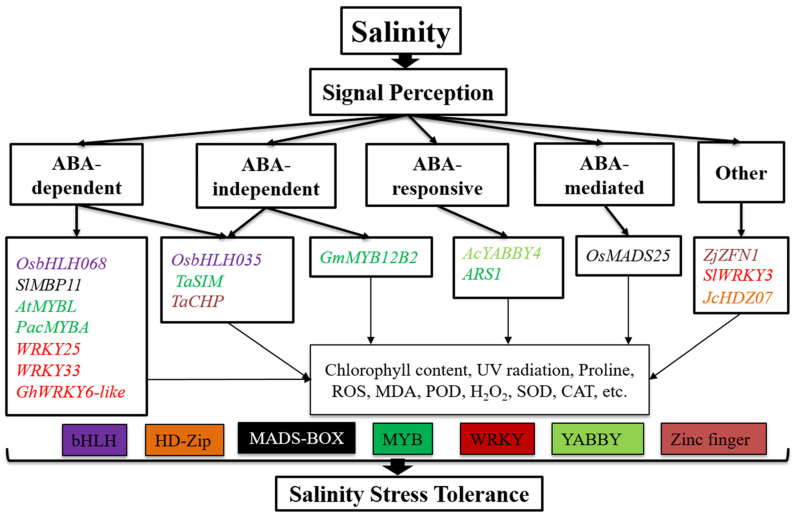
Genes and signaling pathways that regulate salinity stress tolerance. These pathways include the bHLH, HD-Zip, MADS-box, MYB, WRKY, YABBY, and zinc finger. These regulators control salinity stress tolerance through ABA-independent, -responsive, and -mediated pathways, among others, playing an essential role in ROS, chlorophyll content, MDA, POD, SOD, CAT, UV radiation, and proline content. Different text colors represent different transcription factors.

Furthermore, its overexpression in Arabidopsis caused more leaves and a greater leaf area and increased the primary root length [53]. The wheat *TabHLH49* gene is drought stress-related bHLH TF that positively regulates the dehydrin *WZY2* gene and improves drought tolerance in wheat [54]. The rice *OsbHLH068* gene is a member of the bHLH TFs, part of the ABA-dependent pathway, and delayed seed germination and late flowering. *OsbHLH068* overexpression in Arabidopsis resulted in late flowering, delayed seed germination, decreased salt-induced H_2_O_2_ accumulation, increased MDA, and promoted root elongation [55]. The rice *OsbHLH035* bHLH TF is involved in germinating seeds and enabling the recovery of seedlings from salt stress through the ABA-dependent and ABA-independent pathways. After overexpression of the *OsbHLH035* gene, seed germination was delayed, and the average growth of Arabidopsis seedlings recovered after salt stress [56] (Figure 3, Table 1). It is well reported that bHLH TFs play essential roles in gene regulation in many plant species under various abiotic stressors [57]. Arabidopsis *AabHLH35*, a bHLH gene, conferred cold and drought tolerance to *A. andraeanum* and might also help bring tolerance to various abiotic stressors in other ornamental species. *AabHLH35* transgenic Arabidopsis plants better tolerated both cold and drought stress [57]. *AtbHLH112* is a bHLH TF induced by abscisic acid, drought, and salt stress. Arabidopsis plants overexpressing *AtbHLH112* had enhanced salt and drought tolerance, caused by various physiological modulations, including higher proline accumulation and enhanced antioxidant enzyme activities to curb ROS damage [58].

Similarly, in another study, salt and drought stress upregulated the *AtbHLH112* gene, and their knockout mutant phenotype showed late flowering [55]. *EcbHLH57* overexpressing tobacco plants exhibited improved tolerance levels to drought and salt stress. In response to drought stress, transgenic tobacco plants had improved photosynthesis capabilities and higher biomass accumulation. Similarly, EcbHLH57 overexpressing tobacco plants showed minor oxidative damage under salt stress, as lower MDA and H_2_O_2_ levels were observed [59]. The apple (*Malus Domestica*) *MdPIF3* gene is a bHLH TF that plays a critical role in plant growth and development during drought and cold stress. *MdPIF3* overexpression reduced cold tolerance but enhanced drought resistance in apple callus and Arabidopsis [60]. MfbHLH38 is a bHLH gene and has shown a prominent role in improving drought and salt stress tolerance. *MfbHLH38* transgenic Arabidopsis plants have a better water retention ability, osmotic balance, and less oxidative damage. The heterologous expression of *MfbHLH38* in Arabidopsis exhibited better performance, which was observed as higher chlorophyll content, lower MDA level, improved antioxidant enzyme activity, and higher proline and soluble sugar content, under both salt and drought stress, thus enhancing their tolerance. Moreover, the water retention ability of *MfbHLH38* transgenic plants has been greatly improved via stomatal closure due to a higher ABA content and biosynthesis-related gene expression (*NCED3*) under mannitol and ABA treatment [61].

### 2.3. bZIP

The *Poncirus trifoliata ABF* (*PtrABF*) was localized in the nucleus and revealed transactivation action in yeast cells bound to *ABRE*, supporting its role as a TF. Significant levels of *PtrABF* have been stimulated by ABA, low temperature, and dehydration treatments. *PtrABF* overexpression enhanced drought tolerance and dehydration in tobacco by scavenging ROS and modifying the expression of stress-responsive genes [62]. *ABF3, AREB2*, and *AREB1* are excellent TFs that cooperate to complete *ABRE*-dependent ABA-signaling pathways for drought stress tolerance [63]. *OsbZIP72* plays a decisive role in drought resistance through ABA signaling and may help with drought tolerance in rice. *OsbZIP72* is a critical regulator in abiotic stress reactions and ABA signaling transduction pathways [64] (Figure 2, Table 1). A subcellular limitation investigation showed that *TabZIP60* is an atomic restricted protein that initiates TFs. The *TabZIP60* gene is strongly encouraged by treatments with exogenous ABA, salt, polyethylene glycol, and cold. In Arabidopsis, *TabZIP60* gene overexpression fundamentally enhanced resistance to salt and drought stress and expanded plant affectability to ABA in seedling development [65]. The *OsABF2* gene is a constructive controller of ABA signaling and abiotic stress in rice [66]. *OsABF2* has been linked to ABREs (Figure 3), and the homozygous T-DNA insertion mutant of *OsABF2* was susceptible to drought, salinity, and oxidative stress relative to wild-type plants. *OsABF2* functions as a transcription regulator that controls responsive gene expression with abiotic stress through the ABA-dependent pathway [66]. *OsbZIP71* encodes a rice bZIP TF; it is an atomic-limited protein linked to the G-box theme but has no transcriptional movement in yeast or rice protoplasts [67]. OsbZIP71, a bZIP translation factor, may play a vital role in rice ABA-independent drought and salt tolerance [48]. TF *OsbZIP46* directs ABA signaling-mediated drought tolerance in rice by regulating pressure-related genes [68]. ABA and drought pressure activated the *OsbZIP46*-interfacing protein *MODD* (mediator of *OsbZIP46* deactivation and stress), also known as the *Arabidopsis thaliana ABSCISIC ACID-INSENSITIVE5* restricting protein AFP; however, the induction was much slower. *OsbZIP23* is a member of the bZIP TFs. Expression of the *OsbZIP23* gene can cause an adverse effect on stress, including ABA, salt, and drought, while other stress-responsive genes of this family are slightly induced only by one or two of these stressors [69]. *OsABI5* is involved in bZIP TFs and was isolated from *Oryza sativa* L. (Table 1). Expression of the *OsABI5* gene was initiated by high salinity and ABA and downregulated by cold and drought in seedlings. Overexpression of the *OsABI5* gene in rice conserved high sensitivity to salt stress, and *OsABI5* repression enhanced drought stress tolerance and resulted in low rice fertility [70] (Table 1).

### 2.4. DREB

Dehydration-responsive element binding genes (DREBs) are essential plant TFs that control the expression of numerous stress-inducible genes, usually in an ABA-independent manner, and perform a critical role in improving drought and salinity stress tolerance in plants by interacting with a DRE/CRT cis-element present in the promoter region of various genes [71]. *AtDREB1A* overexpression in rice, wheat, groundnut, and tobacco improved drought tolerance and increased the expression of late embryogenic abundant (LEA) genes under greenhouse temperatures [72]. Three DREB homologous genes—*GmDREBa*, *GmDREBb*, and *GmDREBc*—were isolated from soybean; transcription of *GmDREBa* and *GmDREBb* caused drought, salt, and cold stress in the leaves of soybean seedlings (Table 1). Expression of the *GmDREBc* gene was not significantly affected in leaves but prompted by ABA treatment and drought and salt stress [73]. Transgenic Arabidopsis plants with *DREB1* or *DREB2* had improved tolerance to various abiotic stressors, including drought, salt, and freezing [74]. The dehydration responsive element binding (DREB) TF is involved in the plant stress signal transduction pathway. *SbDREB2A* improved abiotic stress tolerance in Escherichia coli; this gene is an A-2 type DREB transferred from the halophyte Salicornia brachiate, and its appearance was encouraged by heat stress, NaCl, and drought [75]. *CBF/DREB1* TFs regulate cool acclimation reactions, and COR TFs (cold-regulated) control gene expression levels, thereby encouraging tolerance to freezing. Thus, changes in CBF/DREB1 genes have enabled many plants to resist environmental stress, mainly freezing [74].

### 2.5. GATA

The GATA gene family is one of the most conserved families of TFs, playing a significant role in different aspects of cellular processes, and their members vary in their expression with a different response to exogenous ABA, drought, and salinity stress. In rice, the *OsGATA23a* gene is a multi-stress responsive TF that increased expression levels under salinity and drought stress. ABA also induced the expression of *OsGATA23a* in different rice varieties [11]. Similarly, the rice *OsGATA16* gene expressed in guard cells and all other plant tissues was induced by ABA treatment but suppressed by drought, cytokinin, and jasmonic acid treatments [76].

### 2.6. HD-Zip

The wheat *TaHDZipI-5* gene, encoding the HD-Zip I TF, was ABA-dependent and regulated the development of drought tolerance. Overexpression of the wheat *TaHDZipI-5* gene improved frost and drought tolerance of transgenic wheat lines. Compared to wild-type (WT) plants, the transgenic wheat lines were short, had delayed flowering, and had decreased grain yield and biomass [77]. The rice *OsTF1L* gene is a crucial regulator of drought tolerance mechanisms, and after overexpression in plants, the drought-inducible stomatal movement was upregulated. Lignin biosynthetic genes revealed a superior drought tolerance at the reproductive growth phase with a higher grain yield than non-transgenic controls under field-drought conditions [78]. The physic nut *JcHDZ07* gene belongs to the HD-Zip family of TFs and is a nuclear-localized protein essential for physiological indices and the necessary regulatory process of plant responses to salinity stress. *JcHDZ07* overexpression in Arabidopsis enhanced the sensitivity of transgenic lines to salt stress.

In contrast, transgenic plants had higher relative electrical leakage and malonaldehyde content than wild-type plants under salinity stress conditions but reduced survival rates, proline content, catalase, and superoxide dismutase activity [79]. Homeodomain–leucine zipper I (HD-Zip) is an essential family of TFs that play crucial roles in responding to various abiotic stressors. *Zmhdz10* overexpression in rice plants caused better performance under drought and salt stress and a better tolerance level to these stressors. Similarly, *Zmhdz10*-overexpressing Arabidopsis plants also conferred salt and drought stress tolerance via differential expression of ABA and stress-responsive gene expression, including *P5CS1*, *RD22*, *RD29B*, and *ABI1*. *Zmhdz10*, a transcriptional regulator, activated the ABA-dependent pathway under drought and salinity stress, thus bringing tolerance to these stressors [10]. *Medicago truncatula MtHB2* encodes a novel stress-responsive HD TF that negatively regulates abiotic stress response mechanisms. In Arabidopsis, transgenic plants expressing *MtHB2* were more sensitive to drought, salt, and freezing stress, had fewer pro and soluble sugars, and had significantly higher MDA and H_2_O_2_ contents than wild-type plants [9]. The rice *Oshox4* gene belongs to the HD-Zip I family, and its overexpression in transgenic lines increased tolerance to drought and salinity stress. The *Oshox4* gene plays an essential role in rice osmatic tolerance and higher yield [80]. *Gshdz4* is an HD-Zip TF in soybean that plays a responsive role in bicarbonate stress and enhances drought and salinity stress tolerance. *Gshdz4* overexpression in Arabidopsis improved transgenic plants’ tolerance to bicarbonate stress via reduced chlorophyll degradation, while their performance was poor under osmotic stress [81]. The Arabidopsis *ATHB17* HD-Zip TF regulated the expression of several photosynthesis-associated nuclear genes involved in the light reaction and *ATSIG5* in response to abiotic stress. *ATHB17* was responsive to ABA and multiple stress treatments and positively modulated the expression of many plastid-encoded genes through the regulation of *ATSIG5*. *ATHB17*-overexpressing plants displayed enhanced stress tolerance, whereas its knockout mutant was more sensitive than the wild-type and played an essential role in protecting plants by adjusting the expression of *PhANGs* and *PEGs* in response to abiotic stress [12].

### 2.7. Homeobox

Homeobox TFs are well-known regulators of plant growth and development [82]. Two stress-responsive homeobox candidate genes, *OsHOX22* and *OsHOX24*, were upregulated under different abiotic stress conditions at various rice growth phases [82]. These gene transcription stages improved in the presence of phytohormones (ABA, auxin, salicylic acid, and gibberellin). *OsHOX24* overexpression affected ABA, abiotic stress, and stress hormones in transgenic Arabidopsis [82]. Many of these genes are engaged in transcriptional control and metabolic pathways, which play the role of homeobox proteins as adverse regulators in abiotic stress response [82].

### 2.8. MADS-Box

**Figure 4:**
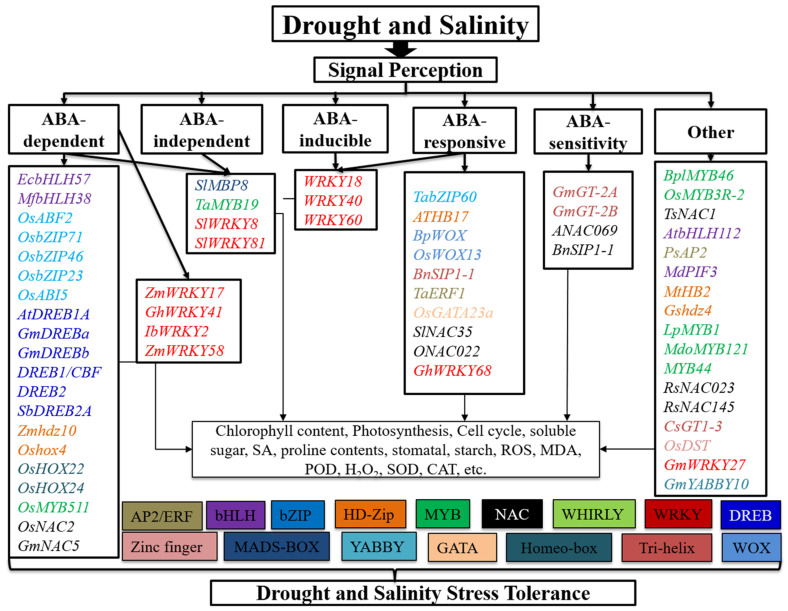
Genes and signaling pathways that regulate drought and salinity stress tolerance. These pathways include the AP2/ERF, bHLH, bZIP, DREB, HD-Zip, Homeo-box, MADS-box, MYB, NAC, Tri-helix, WOX, WHIRLY, WRKY, YABBY, and zinc finger. These regulators control drought and salinity stress tolerance through ABA-dependent, -independent, -responsive, -inducible, and -sensitivity pathways, among others, which play an essential role in photosynthesis, the cell cycle, stomatal development, and ROS, chlorophyll, MDA, POD, SOD, CAT, starch, and proline content. Different text colors represent different transcription factors.

The MADS-box family of TFs are critical regulators of plants and are involved in many biological processes [83]. The *Solanum lycopersicum* agamous-like MADS-box protein *AGL15*-like gene, *SlMBP11*, is a TF that enhances salt stress tolerance, perhaps through an ABA-independent signaling network, and may have applications in the manufacturing of salt-tolerant tomato. *SlMBP11* plays an active role as a stress-responsive TF in the positive regulation of salt stress tolerance utilizing an ABA-independent signaling network and may have significant applications in salt-tolerant tomato design [83]. Overexpression of the rice *OsMADS25* gene in Arabidopsis enhanced salinity tolerance compared to the wild type. The MADS-box transcription factor *OsMADS25* belongs to the ANR1 clade induced by NO^3−^ and plays a crucial role in rice root development [84]. MADS-box TFs are involved in stress reactions. The *SlMBP8* gene containing a MADS-box factor has been cloned from tomato after being expressed in the presence of high salinity, methyl-jasmonic acid, temperature, dehydration, and wounding [85]. *SlMBP8* was downregulated by indole-3-acidic acid (IAA), 1-aminocyclopropane-1-carboxylic acid (ACC), and ABA. *SlMBP8* acts as a negative stress-responsive TF in high-salinity and drought stress signaling pathways and may have important applications in the engineering of salt and drought-tolerant tomato [85] (Figure 4, Table 1).

### 2.9. MYB

Arabidopsis *AtMYB60* is an R2R3-MYB gene expressed in guard cells that is negatively modulated during drought and involved in regulating stomatal movements. The mutant with a T-DNA insertion of *ATMYB60* showed a reduction in the stomatal opening, and the mutation’s effects on water loss and transpiration rate during drought stress [86]. Constructive expression of the rice cold-inducible *OsMYB4* gene in transgenic Arabidopsis plants increased under drought and cold stress and was likely due to the constitutive activation of several stress-inducible pathways. *OsMYB4* gene expression enhanced the stress response in apples [87]. The *GmMYB84* TF from soybean, induced by drought, salt, and ABA, plays a crucial role in ROS homeostasis and control of the abiotic stress response in plants [88]. *GmMYB84* overexpression in soybean enhanced drought stress resistance by increasing the ROS and antioxidant enzyme content, including SOD, POD, and CAT.

Moreover, overexpression led to improved primary growth, high survival rates, and reduced dehydration under drought stress [88]. *OSMYB55* overexpression in maize shows increased plant biomass and reduced leaf damage caused by high-temperature exposure, likely due to increased stress-responsive gene expression [89]. Moreover, it shows reduced initial leaf damage when the chlorophyll content decreases slightly, probably associated with *OsMYB55*-mediated stress tolerance [89]. Similarly, *GaMYB62L* expression in Arabidopsis produced improved drought resistance feedback [90]. *GaMYB85* also encouraged drought tolerance in transgenic Arabidopsis by increasing chlorophyll and free proline material with relatively higher water content [91].

A fascinating novel TF of Arabidopsis, *AtMYBL*, had two estimated DNA-binding domains. The physiological role of R-R-type MYB TFs is unknown in plants [92]. The Arabidopsis *AtMYBL* gene promotes leaf senescence and decreases salt tolerance compared to wild-type and ATMYBL RNA interference lines during subsequent seed growth when subsequent seed growth high-density plants were under stress. *ATMBL* regulates stress sensitivity in protein development [92]. Campos et al. [93] described a salt-sensitive *ars1* mutant phenotype from a single T-DNA insertion in the *ARS1* gene, encoding the *R1-MYB* TF in tomatoes. The T-DNA insertion *ars1* mutant accumulated high Na^+^ in the leaves, accompanied by reduced stomatal conductance and limited transpiration rate, confirming the role of the *ARS1* gene in stomatal movement under salt stress. The sweet cherry *PacMYBA* gene is generally localized to the nucleus and might be induced by ABA. After overexpression of this gene, transgenic Arabidopsis decreased osmotic capability and increased the peroxidase and proline content in response to salt stress [94].

Moreover, *GmMYB12B2* was not induced by the ABA and drought stress response. However, its expression in Arabidopsis caused tolerance to salt stress [95]. Similarly, the MYB TF isolated from wheat had a role in mediating abiotic stress responses. In addition, more recently, *TaSIM* gene overexpression in wheat induced significantly longer roots and further increased the expression level of ABA-dependent (RD22) and ABA-independent (*RD29A*) signaling [96]. This TF is associated with the regulatory system in response to biotic and abiotic stress in plants. The R2R3-MYB factor in *L. purpureus* has also been recognized [97]. *LpMYB1* overexpression in Arabidopsis improved the regeneration of gene transference to drought and salt stress and the capability of genetically modified seedlings in NaCl or ABA. *LpMYB1* is a drought-dependent *R2R3-MYB* factor that builds salt and drought tolerance in Arabidopsis [97]. MYB genes, especially *MdoMYB121*, are enhanced by many stressors. *MdoMYB121* overexpression improved resistance to high salinity, cold stress, and drought in apple plants and transgenic tomatoes. *MdoMYB121* can be used as a target gene in genetic engineering to recover plant tolerance to different abiotic stressors [98]. As a stress protein kinase, the surface protein of MPK3, the Arabidopsis TF, is involved in re-programming pre-stressed *MYB44*. *MYB44* is classified as a phosphorylation-based positive controller of salt stress signaling. *MYB44* conveys a putative transcriptional repression motif. Overexpression of an *MYB44-REP* combination traded salt and drought tolerance [99]. MYB-type proteins take part in various stress responses. The *TaMYB19* gene encodes an R2R3-type MYB protein activated by multiple abiotic stressors in wheat. The expression patterns of *TaMYB19-A*, *TaMYB19-B*, and *TaMYB19-D* were comparable under various stress conditions. The *TaMYB19* protein has an essential role in plant stress tolerance, and adjusting the outflow of this protein may enhance abiotic resilience in crop plants [100]. Plant MYB interpretation factors control various natural processes, for example, separation, improvement, and abiotic stress response. 

*BplMYB46*, an MYB gene from *Betula platyphylla* (birch), is associated with abiotic stress and auxiliary divider biosynthesis. *BplMYB46* enhances salt and osmotic resilience by influencing gene expression, including SOD, POD, and P5CS, to increase reactive oxygen species scavenging and proline. Additionally, *BplMYB46* may help control stomatal openings to diminish water loss [101]. Transgenic *BplMYB46*-overexpressing birch plants showed enhanced salt and osmotic pressure resistance, higher lignin cellulose content, and lower hemicellulose content than the control their potential application in the forestry industry [101] (Table 1). *OsMYB511* is a TF in rice that controls abiotic stress responses and has been activated by exogenous ABA, high temperature, and osmotic pressure. Expression analysis of the *OsMYB511* gene showed high expression at an earlier development stage in rice panicles [102]. A co-articulation investigation uncovered an extra two MYB qualities co-communicated with *OsMYB511*, suggesting that they coordinate direct pressure reactions in rice [102]. *OsMYB3R-2* works in both stress and developmental procedures in rice. Transgenic plants overexpressing *OsMYB3R-2* showed improved cold resistance. The cold treatment initiated the outflow of *OsMYB3R-2*, which encodes a functioning translation factor, and was bound to a mitosis-particular activator cis-component [103]. *GmMYB118* is a soybean gene located in the nucleus that improves tolerance to drought and salt stress by reducing ROS and MDA content and regulates the expression of several stress-associated genes in transgenic Arabidopsis plants. After CRISPR, the *GmMYB118* gene may improve salt stress tolerance in transgenic plants because CRISPR transformed plants displayed reduced drought and salt tolerance compared to control plants [104]. In barley (*Hordeum vulgare* L.), transcripts of *HvMYB1* are upregulated by drought stress in leaves and roots and acting as a mediator of ABA action. Transgenic barley plants that overexpress *HvMYB1* enhanced relative water content and reduced water loss rate, stomatal conductance, and ROS accumulation by constitutively higher ROS scavengers as APX and GPX under drought stress [105].

### 2.10. NAC

The rice gene *OsNAC006* is located in the nucleus, and the knock-out of this gene using the CRISPR-Cas9 system is essential for drought resistance. It is regulated by H_2_O_2_, ABA, heat, cold, and drought treatment. The knockout of *OsNAC006* caused enhanced sensitivity to drought and heat tolerance in rice, which lowered chlorophyll levels, reduced SOD and POD enzyme activities, and increased MDA content [106]. In addition, under the influence of a predominantly root-expressed promoter, *TaRNAC1* improved dehydration resistance, yielding higher biomass, grain production, and root length [107]. *TaRNAC1* is a constitutively and pre-dominantly root-expressed NAC TF. *TaRNAC1* overexpression in wheat roots conferred increased root length and biomass, drought tolerance, and improved grain yield under water limitation [107]. *MfNACsa*, a Medicago falcata lipid-anchored NAC gene, positively regulates plant drought stress resistance by differential expression of oxidation-related, lipid-transported, and localization-related genes [108]. In transgenic tobacco, *SlNAC35* is a protein used to control biotic and abiotic stress resistance. Expression of the *SlNAC35* gene is prompted by salt stress, drought stress, signaling molecules, and bacterial pathogens, suggesting its participation in plant responses to biotic and abiotic stimuli [109]. After overexpression of the *SlNAC35* gene, advanced root development occurs under drought and salt stress [109]. ABA and osmotic stressors, such as drought and high salt, promoted *OsNAC2*, an individual from the NAC translation factor family. *OsNAC2* overexpression reduced high salt and drought tolerance [110]. A microarray showed that numerous ABA-subordinate pressure-related qualities were downregulated in *OsNAC2* overexpression lines. *OsNAC2* directs both abiotic stress reactions and ABA-intervened rice reactions and acts at the intersection between ABA and abiotic stress pathways [110]. NAC proteins are the most significant TFs, and NAC proteins contribute to abiotic stress and plant development regulation. Overexpression of *TsNAC1*, cloned from the halophyte *Thellungiella halophila*, enhanced abiotic stress resistance, particularly salt stress, in *T. halophila* and *Arabidopsis thaliana* and delayed plant development [111]. The *TsNAC1* gene is a crucial TF in abiotic stress resistance and growth [111]. *GmNAC5* is a member of the NAM subfamily and is involved in controlling the shoot apical meristem, hormone signaling, and stress responses in soybean [94]. In addition, *GmNAC5* is stimulated by mechanical wounding and high salt and cold treatments but is not activated by ABA [112]. RsNACs involving layer-bound individuals have been recognized in the radish genome. The *RsNAC023* and *RsNAC080* genes reacted to all stressors, except ABA; however, *RsNAC145* reacted more to heat, salt, and drought. NAC is a strong candidate gene for upcoming studies on improving abiotic stress tolerance in radish [113]. Overexpression of *ANAC069* induced a lower proline content and ROS targeting ability, resulting in enhanced salt and osmotic stress tolerance [114]. After binding to C[A/G]CG[T/G] sequences in the *ANAC069* gene promoter in Arabidopsis, the improvement of proline biosynthesis (P5CS) and antioxidant (POD, SOD, and GST) genes under salt stress was observed [96]. The rice *ONAC022* gene was localized in the nucleus, modulating an ABA-mediated pathway, and a higher survival ratio and less Na^+^ accumulation were observed in roots and shoots in response to drought and salt stress [115].

### 2.11. Trihelix

One of the leading trihelix TF families is the trihelix family TF, also known as the GT factor. Tea (*Camellia sinensis*) genes *CsGT1-3* and *CsGT2-1* belong to the trihelix TFs, which are highly expressed under various abiotic stressors. Salt stress increased the expression of *CsGT1-3* and *CsGT2-1* genes [116]. The soybean *GmGT-2A* and *GmGT-2B* genes are ABA-sensitive trihelix TFs; they increased plant tolerance to abiotic stress when expressed in Arabidopsis [117]. The overexpression of *GmGT-2A* and *GmGT-2B* enhanced resistance to freezing, drought, and salt stress in transgenic Arabidopsis [117]. The trihelix family genes are involved in light and other developmental processes, but their characteristics are generally unclear. The *BnSIP1-1* protein is focused on the nucleus. Overexpression of *BnSIP1-1* enhanced seed germination when exposed to osmotic stress, ABA, and salt. *BnSIP1-1* is likely involved in ABA signaling and synthesis and osmotic and salt stress responses [118]. A rice *OsGTγ* family member, *OsGTγ-2*, directly interacted with the GT-1 element in the *OsRAV2* promoter. OsGTγ-2 specifically targeted the nucleus and was mainly expressed in roots, sheaths, stems, and seeds, and it was induced by salinity, osmotic and oxidative stresses, and ABA [119]. *Arabidopsis thaliana AtGT2L* and rice *OsGTγ-1* were induced by salt, drought, cold stress, and ABA treatment [120]. The function of *AST1* was characterized in response to salt and osmotic stress and showed transcriptional activation activity; its expression was induced by osmotic and salt stress [121].

### 2.12. WHIRLY 

*WHIRLY* is a TF family involved in biotic and abiotic stress responses, but its biological function remains unclear. *SlWHY2*, an individual from the *WHIRLY* family, was isolated from tomatoes [122]. Overexpression of the *SlWHY2* gene in tobacco improved drought stress tolerance, controlled mitochondrial gene transcription, and balanced mitochondrial metabolism. The *SlWHY2* gene is a positive controller for plants exposed to biotic and abiotic stress [122]. 

### 2.13. WOX

WOX (WUSCHEL-related homeobox) is a plant TF linked to plant development and stress responses. The paper mulberry *BpWUS* gene is an ABA-responsive gene associated with the stem, root, and apical bud. *BpWOX5* and *BpWOX7* controlled the root tip, and three *BpWOXs* controlled leaf enlargement [123]. *BpWOX9* and *BpWOX10* were promoted by indole-3-acidic (IAA) or jasmonic (JA), while five phytohormones repressed *BpWOX2*. Most *BpWOX* genes were receptive to drought, salt, cold, and cadmium (CdCl_2_) [123]. The rice *WOX13* gene belongs to the *WOX* subfamily of TFs and is ABA-responsive, essential for flower improvement, contains proteins, and is involved in drought and salinity stress. *OsWOX13* was involved in the regulation of vegetative organs, flowers, and seeds. *OsWOX13* caused early flowering and stress responses. *OsWOX13* overexpression resulted in early flowering and showed an extensive spectrum of effects on biological processes, such as abiotic and biotic stress, after drought and salinity stress [124].

### 2.14. WRKY

*WRKY46*, *WRKY54*, and *WRKY70* are three WRKY TFs in Arabidopsis that are correlated with drought response and BR-mediated plant development. The mutants of *wrky46*, *wrky54*, and *wrky70* had altered plant development, controlled drought, and promoted BR-mediating gene expression and drought response genes in RNA sequencing analysis [125]. Sun et al. [126] found that the activated expression of the group III member protein *AtWRKY53* modulated stomatal movement, improving starch metabolism and functioning as osmoregulation by decreasing H_2_O_2_ content, contributing negatively to controlling dehydration tolerance. Likewise, in rice, *OsWRKY47* imparted drought stress tolerance [127]. *OsWRKY47* expression was caused by drought stress in plants, and their mutants showed higher susceptibility to drought and decreased yield, whereas *OsWRKY47* plants were more tolerant [127]. Expression of the *Glycine soja GsWRKY20* gene improved drought tolerance and modified ABA signaling. After *GsWRKY20* overexpression in Arabidopsis, plants had reduced sensitivity to ABA, stomatal closure during seed germination, and early seedling growth, exhibiting a greater tolerance to drought stress [128]. Past microarray investigations of Arabidopsis roots recognized two WRKY TFs (*WRKY25* and *WRKY33*) among the transcripts that expanded NaCl treatment. *WRKY33* is not flexible in any situation affecting *WRKY25* null mutants’ stress, indicating functional redundancy with null mutants and two-fold mutants and promoting NaCl sensitivity. When *WRKY25* or *WRKY33* were overexpressed in Arabidopsis, plants had NaCl tolerance [129]. Likewise, in cotton, the *GhWRKY6* gene was observed to target ROS and stimulate the ABA signaling pathways, consequently improving salt tolerance in Arabidopsis.

In contrast, *GhWRKY6* gene silencing by virus-induced gene silencing (VIGS) in cotton improved susceptibility to abiotic stress [130]. In addition, a recent study found that *SlWRKY3* protein overexpression encouraged physiological indices correlated with photosynthesis, increased leaf aggregation of K^+^ and Ca^2+^, and decreased sodium and proline content [131]. WRKY TFs are linked with biotic and abiotic stress in plant reactions. Arabidopsis TFs *WRKY18*, *WRKY40*, and *WRKY60* cooperate functionally and physically in plant resistance responses [132]. The three WRKY genes are associated with plant reactions to ABA and abiotic stress. Overexpression of distinctive mutants for WRKY genes showed that *WRKY18* and *WRKY60* positively affected plant ABA to restrict seed germination and root development [132]. *WRKY18* and *WRKY60* genes were affected by abiotic stress in germination, plant sensitivity to ABA, and growth assays. *WRKY18* and *WRKY40* were quickly induced after ABA treatment, whereas *WRKY60* was not rapidly induced [113].

Furthermore, the maize *ZmWRKY17* gene in Arabidopsis decreased ABA sensitivity, as shown by healthy green cotyledons and longer roots in response to exogenous ABA application, and increased plant sensitivity to salinity stress [133]. *GhWRKY41* [134] and *GhWRKY68* are two other cotton WRKY genes that positively regulate salt and drought stress resistance by affecting physiological indices, including stomatal closer and ROS accumulation in transgenic *Nicotiana benthamiana* [135]. *MtWRKY76* induced abiotic stress-responsive genes associated with the ASR protein in *M. truncatula*, resulting in increased drought and salt tolerance [136]. Genetic research in soybean revealed that *GmWRKY27* improves salt and drought tolerance, which was confirmed by the proline and ROS content [137]. The sweet potato *IbWRKY2* gene was found in the nucleus, and NaCl and ABA induced its expression. In addition, Arabidopsis overexpressing *IbWRKY2* demonstrated improved drought and salt tolerance. The content of ABA and proline and the activity of SOD were higher in transgenic plants after drought and salt treatments, while the contents of MDA and H_2_O_2_ were lower [138]. Similarly, *ZmWRKY58* also played an essential role in response to drought and salt stress in rice. Overexpression of *ZmWRKY58* in rice resulted in delayed germination and inhibited post-germination development [139]. In tomato (*Solanum lycopersicum*), *SlWRKY8* protein was localized to the nucleus, and a positive regulator in plant immunity against pathogen infection and plant response to drought and salt stresses through ABA-dependent and ABA-independent pathways. Overexpression of *SlWRKY8* promoted the activities of ROS-scavenging enzymes and proline contents [140]. In tomatoes, the transcript of *SlWRKY81* is involved in the regulation of ABA-mediated and acts as a negative regulator for drought tolerance by modulating stomatal movement. Overexpression of *SlWRKY81* enhances tomato tolerance to drought and promotes ABA content, stomatal closure, and accumulation of H_2_O_2_ in the guard cells [141]. Similarly, Ahammed et al. [142,143] reported that the *SlWRKY81* TF inhibits stomatal closure by reducing nitric oxide accumulation in the guard cells and is closely associated with an increased proline content in tomato leaves compared with non-silenced plants of tomatoes under drought.

### 2.15. YABBY

YABBY plays a vital monitoring role in lateral organ development. The pineapple *AcYABBY* gene, after overexpression in Arabidopsis, displayed a small root under NaCl treatment, representing the adverse effect of *AcYABBY4* on plant resistance to salt stress [144]. *GmYABBY10* might be a negative regulator of plant tolerance to drought and salt stress. The *GmYABBY10* protein was mainly localized in the membranes and cytoplasm, which are more sensitive to drought, salt, and ABA stress. *GmYABBY10* played an essential role in drought and salt resistance in Arabidopsis, and wild-type seeds had higher than *GmYABBY10* transgenic seeds under both PEG and NaCl treatment. Simultaneously, wild-type seedlings’ root length and root surface were more extensive than *GmYABBY10* transgenic seedlings [145].

### 2.16. Zinc Finger

The *Chrysanthemum morifolium BBX24* gene encoding a zinc finger TF was mainly associated with flowering time and stress tolerance. Transgenic lines with suppressed expression of *Cm-BBX24* (*Cm-BBX24-RNAi*) showed early flowering compared to wild-type plants and exhibited decreased tolerance to drought and freezing stress in chrysanthemum, in part, by influencing GA biosynthesis [146]. The gene from the *CCCHZF* rice family, *OsC3H10*, primarily expressed in plants, consequently causes a rapid decline during seed imbibition; moreover, the expression of *OsC3H10* was induced by drought high salinity and ABA [147]. *OsC3H10* regulated drought resistance by modulating stress-related gene expression involving various drought-tolerant pathways. However, root-specific overexpression of *OsC3H10* was inadequate to cause drought tolerance, whereas the plant overall had increased drought tolerance [147]. Overexpression of the zinc finger protein *ZAT18*, expressed in the roots, silica, and rosette plants, resulted in drought tolerance in Arabidopsis, with more minor leaf water losses, lower ROS quality, higher leaf water content, and higher antioxidant enzyme activity after drought treatment relative to the wild-type [148] (Figure 2, Table 1). Several genes of zinc finger proteins are involved in playing essential roles in salt stress. The *Zoysia japonica ZjZFN1* gene is a zinc finger TF that plays a critical role in improved seed germination and enhanced plant salt tolerance in Arabidopsis. Plant variation also improved with salinity stress with improved green cotyledons and growth status under salinity stress. *ZjZFN1*-overexpressing plants revealed that *ZjZFN1* might be a transcriptional activator of changeable stress-responsive pathways, including α-linolenic acid metabolism, phenylalanine metabolism, and phenylpropanoid biosynthesis pathways [149]. The bread wheat *TaCHP* gene belongs to the zinc finger family, which is essentially expressed in the roots of seedlings at the three-leaf stage. *CHP* was reduced by the imposition of salinity or drought stress and the exogenous supply of ABA [150] (Figure 3, Table 1). Using CRISPR-Cas9 mediated genome editing in rice (*OsDST*), the *DST* gene increased drought and salinity stress tolerance and improved crop production. The *DST* mutant was first produced in rice, and stomatal density was associated with reducing stomatal development genes in the *DST* mutant [151] (Figure 4, Table 1).

### 2.17. Other

AITRs, as a family of novel TFs, play a role in regulating plant responses to ABA, drought, and salinity stress. Using CRISPR/Cas9 to target six *AITR* genes (*aitr123456*) reduced sensitivity to ABA and enhanced tolerance to drought and salinity in the Arabidopsis mutant, but plant growth, development, and response to pathogen infection remained unaffected in the mutants [152].

## 3. Conclusions and Future Research Priorities

Plants cannot escape environmental pressures due to their sessile existence, but they have developed strategies to counteract the adverse effects of stress. Plant endogenous development programs, for example, use physiological and metabolic modifications to help plants cope with unfavorable environmental factors, including salinity and drought. Plant production and productivity may be negatively impacted by the failure to respond to adverse environmental factors, resulting in a substantial reduction in yield. This review covered the current knowledge on drought and salt stress genes and focused on the various TFs involved in drought and salt stress, showing the apparent link with ABA-dependent and -independent pathways. Tremendous improvements have been made to understand the molecular mechanisms controlling drought and salinity stress tolerance in recent years. Several regulatory pathways have been identified for drought and salinity tolerance in different plants (apple, Arabidopsis, chrysanthemum, finger millet, maize, pineapple, rapeseed, rice, soybean, tea, tomato, and wheat) using genetic engineering and CRISPR/Cas9 for genome editing.

**Figure 5:**
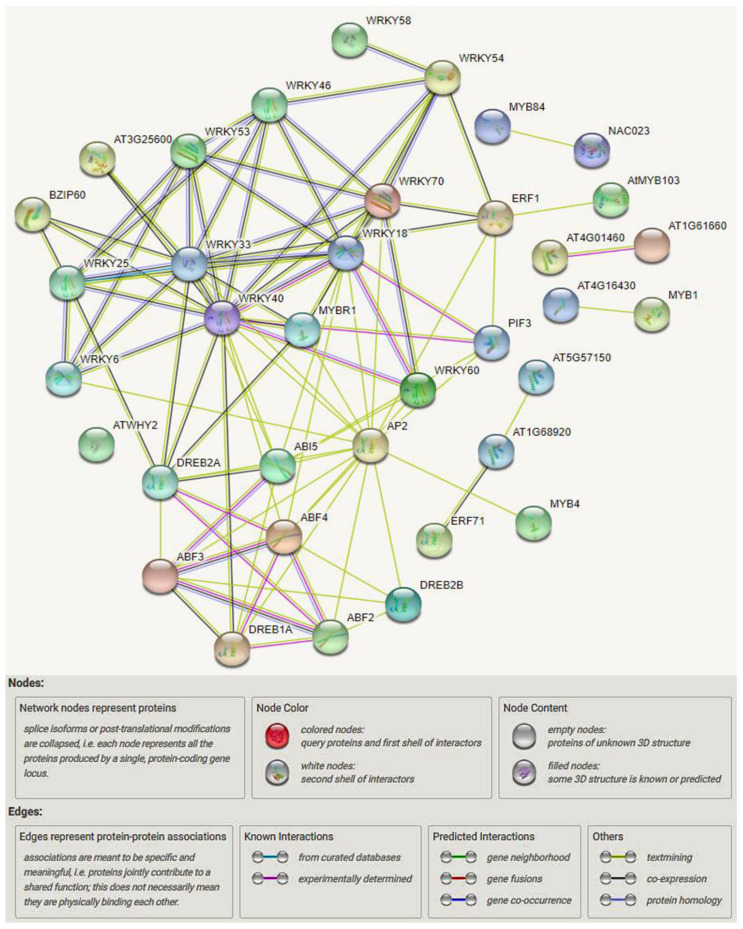
Genetic regulatory network constructed for drought and salinity tolerance genes. The figure shows different interactions, such as gene neighborhood, fusions, co-occurrence, text mining, co-expression, and protein homology. For example, green represents the gene neighborhood, red represents gene fusion, blue represents gene co-occurrence, yellow represents text mining, and black represents co-expression.

Similar to Hussain et al. [42], we revealed complex genetic regulatory networks (Figure 5) based on examining current drought and salinity stress tolerance knowledge in Arabidopsis and other plant species. Several genetic and signaling pathways that determine drought and salinity stress tolerance are well known, including AP2/ERF, bHLH, bZIP, DREB, GATA, HD-Zip, Homeo-box, MADS-box, MYB, NAC, Tri-helix, WHIRLY, WOX, WRKY, YABBY, and zinc finger (Table 1). Interestingly, many of these genes have a conserved function in drought and salinity stress, and their pathways are ABA-dependent, -independent, -induced, -responsive, -mediated, and -sensitive. In addition, many of these genes have similar functions in drought and salinity stress, which belong to ABA-dependent, -independent, -inducible, -responsive, and -sensitive pathways that regulate the cell and ROS scavenging. Their TFs are bHLH, bZIP, Homeo-box, DREB, MYB, NAC, HD-Zip, MADS-box, WOX, and WRKY. More importantly, many of the drought and salinity stress-responsive genes have expression activity and additional effects on other organs, such as flowering time (*Cm-BBX24*) and yield (*OsCML16*, *OsERF71*, *TaHDZipI-5*, *OsTF1L*, and *Oshox4*), indicating a cooperative regulation. These results strongly suggest the conserved function of these genes in regulating drought and salinity stress tolerance among different plant species. They can be targeted for the molecular improvement of drought, and salinity stress tolerance through genetic engineering and genome editing approaches, such as CRISPR/Cas.

Most importantly, with these approaches to TFs, the future development of drought- and salinity-resistant plants with improved yields and reduced off-target effects will become a reality. In the future, a combination of modern biotechnologies, such as microarray, proteomic genome editing, genomics, genome-wide association, -omics, and bioinformatics, will accelerate the identification of the regulators of drought and salinity stress responses and different genes and signaling pathways. In conclusion, it is necessary to collaborate to convey this science-based benefit to farmers to deliver a food supply adequate to eliminate world hunger. The link between transcription and phytohormones was further identified, as well as their signaling pathways. Candidate genes that regulate and target different phytohormones can consequently mitigate drought and salinity stress. Tolerance to these stressors in crop breeding is mainly unknown.

## Figures and Tables

**Table 1:** The genes involved in drought and salinity stress tolerance in different plants.

Gene Name	TFs	Function	Expression	Species	References
*OsERF48*	AP2/ERF	Enhances root growth	Seedlings roots	Rice	[46]
*OsERF71*	AP2/ERF	Cell wall modification, root structure	Root meristem	Rice	[47]
*SHN1*	AP2/ERF	Activates Wax Biosynthesis	Flower	Arabidopsis	[48]
*SHN2*	AP2/ERF	Activates Wax Biosynthesis	Flower	Arabidopsis	[48]
*SHN3*	AP2/ERF	Activates Wax Biosynthesis	Flower	Arabidopsis	[48]
*OsERF922*	AP2/ERF	Modulation of the ABA levels	Shoot	Rice	[49]
*PsAP2*	AP2/ERF	Regulate the level of RNS and ROS	Leaves, floral bud, root	*Papaver somniferum*	[43]
*TaERF1*	AP2/ERF	Stress signal transduction pathways	Leaf	Wheat	[50]
*AtbHLH68*	bHLH	Regulation of lateral root elongation	Shoot and root	Arabidopsis	[51]
*FtbHLH3*	bHLH	Activating the antioxidant system	Root, stem, flower, and leaves	*Fagopyrum tataricum*	[52]
*PebHLH35*	bHLH	Regulating stomatal density and aperture	Root and leaf	Arabidopsis	[53]
*TabHLH49*	bHLH	Regulates dehydrin WZY2 gene expression	Leaves, stem and roots	wheat	[54]
*OsbHLH068*	bHLH	Control flowering	Leaves and aerial tissues	Arabidopsis	[55]
*OsbHLH035*	bHLH	Reduces ABA levels	Germinating seeds, seedlings	Rice	[56]
*AabHLH35*	bHLH	Improved tolerance to drought stress	Leaf	*Anthurium andraeanum*	[57]
*AtbHLH112*	bHLH	Proline biosynthesis and ROS scavenging	Root, leaves	Arabidopsis	[58]
*EcbHLH57*	bHLH	Improved root growth	Leaf, root	Finger millet	[59]
*MdPIF3*	bHLH	Positively regulates the drought resistance	Root	*Malus domestica*	[60]
*MfbHLH38*	bHLH	Regulating osmotic balance	Leaves, roots	*Myrothamnus flabellifolia*	[61]
*PtrABF*	bZIP	Scavenging ROS and enhances dehydration	Leaves	*Poncirus trifoliate*	[62]
*ABF3*	bZIP	Activate target genes in ABA signaling	Root	Arabidopsis	[63]
*AREB1*	bZIP	Activate target genes in ABA signaling	Root	Arabidopsis	[63]
*AREB2*	bZIP	Activate target genes in ABA signaling	Root	Arabidopsis	[63]
*OsbZIP72*	bZIP	Positive regulator of ABA response	Seedlings	Rice	[64]
*TabZIP60*	bZIP	Increased plant sensitivity to ABA	Spikes, leaves, stems	Wheat	[65]
*OsABF2*	bZIP	Positive regulator of ABA signaling	Various rice tissues	Rice	[66]
*OsbZIP71*	bZIP	Important role in ABA-mediated	Root, shoot	Rice	[67]
*OsbZIP46*	bZIP	Negatively regulate ABA signaling	Leaf	Rice	[68]
*OsbZIP23*	bZIP	Improved sensitivity to ABA	Leaves, root, shoot	Rice	[69]
*OsABI5*	bZIP	Low fertility	Mature pollen	Rice	[70]
*AtDREB1A*	DREB	Higher proline and SOD activity	Leaves	Arabidopsis	[72]
*GmDREBa*	DREB	Transcriptional activation ability	Leaves, seedlings	Soybean	[73]
*GmDREBb*	DREB	Transcriptional activation ability	Leaves, seedlings	Soybean	[73]
*DREB1/CBF*	DREB	Acquisition of stress tolerance	Seed maturation	Arabidopsis	[74]
*DREB2*	DREB	Acquisition of stress tolerance	Seed maturation	Arabidopsis	[74]
*SbDREB2A*	DREB	Response to stress	Leaves and root	*Salicornia brachiata*	[75]
*OsGATA23a*	GATA	Response to environmental signals	Seedling, stem	Rice	[11]
*OsGATA16*	GATA	Enhanced drought tolerance	Panicles, guard cells	Rice	[76]
*TaHDZipI-5*	HD-Zip	Delayed flowering and a grain yield decrease	Flowers and grains	Wheat	[77]
*OsTF1L*	HD-Zip	Lignin biosynthesis and stomatal closure	Root, shoot, flower	Rice	[78]
*JcHDZ07*	HD-Zip	Changes in physiological indexes	Roots, leaves, seeds	Arabidopsis/Nut	[79]
*MtHB2*	HD-Zip	Negative role in regulation of abiotic stress	Pods, leaves, root, stem	*Medicago truncatula*	[9]
*Zmhdz10*	HD-Zip	ABA signal transduction pathway	Root, stem, tassels, ears, leaf	Maize	[10]
*ATHB17*	HD-Zip	Alleviating the damage to chloroplast	Root, leaves	Arabidopsis	[12]
*Oshox4*	HD-Zip	Controlling ABA signal perception	Leaves	Rice	[80]
*Gshdz4*	HD-Zip	Positively regulates bicarbonate	Leaves, stem, root	Soybean	[81]
*OsHOX22*	Homeobox	Higher sensitivity to ABA and hormones	Root, fresh weight	Rice	[82]
*OsHOX24*	Homeobox	Higher sensitivity to ABA and hormones	Root, fresh weight	Rice	[82]
*SlMBP11*	MADS-box	Higher chlorophyll content, higher MDA	Root and shoot	Arabidopsis	[83]
*OsMADS25*	MADS-box	Higher proline contents, MDA	Seedling, shoot and root	Rice	[84]
*SlMBP8*	MADS-box	Negative regulator in stress response	Root, sepals and fruits	Tomato	[85]
*AtMYB60*	MYB	Stomatal Movements	Seedling, stem, leaves, flower	Arabidopsis	[86]
*OsMYB4*	MYB	Improved physiological and biochemical adaptation	Leaves, root, stem, flower, seed	Rice/Transgenic Apple	[87]
*GmMYB84*	MYB	Improves drought stress response and promotes root growth	Root and flower	Soybean	[88]
*OsMYB55*	MYB	Encoding proteins involved in general defense responses and abiotic stress	Seedlings	Rice/Maize	[89]
*GaMYB62L*	MYB	Enhanced the expression of ABA	Root and leaves	Arabidopsis	[90]
*GaMYB85*	MYB	Reduced stomatal density, with greater stomatal size	Seedlings	Cotton	[91]
*AtMYBL*	MYB	Promoting leaf senescence	Leaves	Arabidopsis	[92]
*ARS1*	MYB	Stomatal closure	Root, flower, leaves	Tomato	[93]
*PacMYBA*	MYB	Pathogen resistance	Leaf	Sweet cherry	[94]
*GmMYB12B2*	MYB	Regulates UV radiation	Seedlings	Soybean	[95]
*TaSIM*	MYB	Improve crop resistance to salt stresses	Root, leaf, and stem	Wheat	[96]
*LpMYB1*	MYB	Improve the drought and salt tolerance	Seedling, root, seeds	*Lablab purpureus*	[97]
*MdoMYB121*	MYB	Roles in secondary metabolism	Seed germination, seedling	Tomato/Apple	[98]
*MYB44*	MYB	Oxidative damage and hypersensitivity	Leaves, seedlings	Arabidopsis	[99]
*TaMYB19*	MYB	Leads to improved stress tolerance	Root, seedlings	Wheat	[100]
*BplMYB46*	MYB	Affects secondary cell wall deposition	Stem, leaves, root	*Betula platyphylla*	[101]
*OsMYB511*	MYB	Panicle development	Panicles at an earlier stage	Rice	[102]
*OsMYB3R-2*	MYB	Mediated by regulating the cell cycle	Seedling	Rice	[103]
*GmMYB118*	MYB	Reducing the contents of ROS and MDA	Root	Soybean	[104]
*HvMYB1*	MYB	Acting as a mediator of ABA action	Roots and leaves	Barley	[105]
*OsNAC006*	NAC	Responses to stimuli, cofactor binding	Stems and leaves	Rice	[106]
*TaRNAC1*	NAC	Enlargement of the root system	Root	Wheat	[107]
*MfNACsa*	NAC	Oxidation-reduction and lipid transport	Root and leaves	*Medicago falcata*	[108]
*SlNAC35*	NAC	Involving auxin and SA signaling	Roots	Tomato	[109]
*OsNAC2*	NAC	Regulates both abiotic stress responses and ABA-dependent	Root and leaves	Rice	[110]
*TsNAC1*	NAC	Regulates the expansion of cells	Root, mature tissues, shoot	*T. halophila*	[111]
*GmNAC5*	NAC	Involved in seed development and abiotic stress responses	Roots and immature seeds	Soybean	[112]
*RsNAC023*	NAC	Reacted to all stresses except ABA	Roots, flowers, and leaves	Radish	[113]
*RsNAC145*	NAC	Reacted to all stresses except ABA	Root, flower, and leaves	Radish	[113]
*ANAC069*	NAC	Decreased ROS scavenging capability and proline biosynthesis	Leaves, stems, siliques	Arabidopsis	[114]
*ONAC022*	NAC	Modulating an ABA-mediated pathway	Seedling and panicles	Rice	[115]
*CsGT1-3*	Tri-helix	Stress tolerance	Leaves	Tea Plant	[116]
*CsGT2-1*	Tri-helix	Stress tolerance	Leaves	Tea Plant	[116]
*GmGT-2A*	Tri-helix	Regulate plant stress responses	Stem, pods	Soybean	[117]
*GmGT-2B*	Tri-helix	Regulate plant stress responses	Stem, pods	Soybean	[117]
*BnSIP1-1*	Tri-helix	Roles in ABA synthesis and signaling	Roots, stems, leaves, pollens	*Brassica napus*	[118]
*OsGTγ-2*	Tri-helix	Regulating salinity adaptation	Roots, stems and seeds	Rice	[119]
*AtGT2L*	Tri-helix	Interacts with calcium/calmodulin	Flowers and leaves	Arabidopsis	[120]
*AST1*	Tri-helix	Reduced ROS accumulation	Leaves, stems, and anthers	Arabidopsis	[121]
*SlWHY2*	WHIRLY	Reducing ROS accumulation	Pollens	Tomato	[122]
*BpWOX*	WOX	Plant development and stress responses	Apical bud, stem, and root	*Paper mulberry*	[123]
*OsWOX13*	WOX	Triggers early flowering	Leaves	Rice	[124]
*WRKY46*	WRKY	BR-regulated plant growth	Leaves	Arabidopsis	[125]
*WRKY54*	WRKY	BR-regulated plant growth	Leaves	Arabidopsis	[125]
*WRKY70*	WRKY	BR-regulated plant growth	Leaves	Arabidopsis	[125]
*AtWRKY53*	WRKY	Mediating stomatal movement	Guard cells	Arabidopsis	[126]
*OsWRKY47*	WRKY	Reduction in photosynthesis and high yields	Leaves	Rice	[127]
*WRKY20*	WRKY	Regulates ABA signaling	Seedlings	Soybean	[128]
*WRKY25*	WRKY	Increasing sensitivity to ABA	Leaves, siliques, flower, root	Arabidopsis	[129]
*WRKY33*	WRKY	Increasing sensitivity to ABA	Leaves, siliques, flower, root	Arabidopsis	[129]
*GhWRKY6-like*	WRKY	Activating the ABA signaling pathway, scavenging of ROS	Roots, stem, leaves, flowers, and anthers	Cotton	[130]
*SlWRKY3*	WRKY	Regulation of senescence related process	Leaves and mature fruit	Tomato	[131]
*WRKY18*	WRKY	Plant defense and stress responses	Seed germination and root	Arabidopsis	[132]
*WRKY40*	WRKY	Plant defense and stress responses	Seed germination and root	Arabidopsis	[132]
*WRKY60*	WRKY	Plant defense and stress responses	Seed germination and root	Arabidopsis	[132]
*ZmWRKY17*	WRKY	Decreased ABA sensitivity	Tassels, Leaf, root	Maize	[133]
*GhWRKY41*	WRKY	Enhanced stomatal closure	Stomata	Cotton	[134]
*GhWRKY68*	WRKY	Regulating ABA signaling	Leaf	Cotton	[135]
*MtWRKY76*	WRKY	Increased salt and drought tolerance	Root, seedling	*Medicago truncatula*	[136]
*GmWRKY27*	WRKY	Improvements in stress tolerance	Root, cotyledons	Soybean	[137]
*IbWRKY2*	WRKY	Enhancing the tolerance to abiotic stress	Seedling, leaves, germination	Sweet potato	[138]
*ZmWRKY58*	WRKY	Positive regulator of stress tolerance	Root, leaf, germination	Rice/Maize	[139]
*SlWRKY8*	WRKY	Resistance to pathogen infection	Stem, roots, flowers	Tomato	[140]
*SlWRKY81*	WRKY	Regulator of stomatal closure	Leaves	Tomato	[141,142,143]
*AcYABBY4*	YABBY	Important role in response to ABA	Sepal and petal	Pineapple	[144]
*GmYABBY10*	YABBY	Highly sensitive in drought	Seedling, root, germination	Soybean	[145]
*Cm-BBX24*	Zinc finger	Modulating gibberellin biosynthesis	Root, leaves, stem	*Chrysanthemum*	[146]
*OsC3H10*	Zinc finger	Response to drought	Seeds	Rice	[147]
*ZAT18*	Zinc finger	Positive drought stress regulator	Stems, siliques, leaves	Arabidopsis	[148]
*ZjZFN1*	Zinc finger	Stress responses in seed germination	Leaf, stem, root	*Zoysia japonica*	[149]
*TaCHP*	Zinc finger	Enhances stress tolerance	Roots, leaf	Wheat	[150]
*OsDST*	Zinc finger	Stomatal density	Flag leaf	Rice	[151]
*OsRR22*	Other	Enhanced the tolerance to salinity	Shoot	Rice	[152]

## Data Availability

No supplementary data is available.

## Abbreviations

ABA: Abscisic Acid; AP2/ERF, APETALA2/ETHYLENE RESPONSIVE FACTOR; AREB/ABF, RESPONSIVE ELEMENT BINDING FACTORS; bHLH, basic/helix-loop-helix; BR, brassinosteroid; bZIP, Basic leucine zipper; CBFs, C-repeat Binding Proteins; CBLs, Calcineurin-B-like proteins; CDPKs, Calcium-dependent protein kinases; CRISPRs)/Cas, clustered regularly interspaced short palindromic repeats; ChIP-seq, Chromatin immunoprecipitation sequencing; CIPK, CBL-interacting protein kinase; DAP-seq, DNA affinity purification sequencing; DREBs, Dehydration Responsive Element Binding Proteins; GRNs, Gene regulatory networks; H_2_O_2_, Hydrogen Peroxide; HD-Zip, Homeodomain-leucine zipper; MAPK, Mitogen-activated protein kinase; MDA, malondialdehyde; PKs, Protein kinases; POD/POX, Peroxidase; PP2Cs, Protein phosphatase 2Cs; RNS, reactive nitrogen species; PPs, protein phosphatases ROS, Reactive Oxygen Species; SA, salicylic acid; SOD, Superoxide dismutase; TALENs, transcriptional activator-like nucleases; TFs, transcription factors; WOX, WUSCHEL-related homeobox; WT, Wild-type; ZFNs, zinc-finger nucleases.
